# Co-use of MDMA with psilocybin/LSD may buffer against challenging experiences and enhance positive experiences

**DOI:** 10.1038/s41598-023-40856-5

**Published:** 2023-08-22

**Authors:** Richard J. Zeifman, Hannes Kettner, Broc A. Pagni, Austin Mallard, Daniel E. Roberts, David Erritzoe, Stephen Ross, Robin L. Carhart-Harris

**Affiliations:** 1 NYU Langone Center for Psychedelic Medicine, Department of Psychiatry, NYU Grossman School of Medicine, 1 Park Avenue, New York, NY 10016 USA; 2 Centre for Psychedelic Research, Department of Brain Sciences, Faculty of Medicine, Imperial College London, London, UK; 3 Psychedelics Division, Neuroscape, University of California, San Francisco, USA

**Keywords:** Human behaviour, Combination drug therapy, Clinical pharmacology

## Abstract

Psilocybin and lysergic acid diethylamide (LSD) experiences can range from very positive to highly challenging (e.g., fear, grief, and paranoia). These challenging experiences contribute to hesitancy toward psychedelic-assisted psychotherapy among health care providers and patients. Co-use of 3,4-Methylenedioxy methamphetamine (MDMA) with psilocybin/LSD anecdotally reduces challenging experiences and enhances positive experiences associated with psilocybin/LSD. However, limited research has investigated the acute effects of co-use of MDMA and psilocybin/LSD. In a prospective convenience sample (*N* = 698) of individuals with plans to use psilocybin/LSD, we examined whether co-use of MDMA with psilocybin/LSD (*n* = 27) is associated with differences in challenging or positive experiences. Challenging experiences were measured using the Challenging Experiences Questionnaire and positive experiences were measured using the Mystical Experience Questionnaire and single-item measures of self-compassion, compassion, love, and gratitude. Potentially confounding variables were identified and included as covariates. Relative to psilocybin/LSD alone, co-use of psilocybin/LSD with a self-reported low (but not medium–high) dose of MDMA was associated with significantly less intense total challenging experiences, grief, and fear, as well as increased self-compassion, love and gratitude. Co-use of psilocybin/LSD and MDMA was not associated with differences in mystical-type experiences or compassion. Findings suggest co-use of MDMA with psilocybin/LSD may buffer against some aspects of challenging experiences and enhance certain positive experiences. Limitations include use of a convenience sample, small sample size, and non-experimental design. Additional studies (including controlled dose–response studies) that examine the effects and safety of co-administering MDMA with psilocybin/LSD (in healthy controls and clinical samples) are warranted and may assist the development of personalized treatments.

Classic psychedelics, such as psilocybin and lysergic acid diethylamide (LSD), are non-selective 5-HT2A receptor agonists with therapeutic potential for treating psychiatric disorders and mental health concerns (for a review, see^1^). Classic psychedelics show a fairly strong safety profile, including minimal adverse effects, toxicity, and potential for abuse^2–5^. A primary concern associated with classic psychedelics relates to their alteration of consciousness^3^, which can range from highly positive ‘peak’ experiences^6,7^ to psychologically challenging experiences (often referred to as “bad trips”^8,9^), such as grief, paranoia, and fear^10^.

Challenging experiences following use/administration of classic psychedelics have been reported in both controlled (e.g., clinical trials) and uncontrolled (e.g., ritual or recreational use) studies (e.g.,^11–19^). For instance, in a clinical trial in which individuals with major depressive disorder received two doses of psilocybin alongside psychotherapy, 65% of individuals described one of their psilocybin experiences as one of the five most psychologically challenging experiences of their life and 25% of individuals described it as the single most psychologically challenging experience of their life^13^. Furthermore, across their two psilocybin experiences in this clinical trial^13^, 92% of individuals reported that they felt like crying (although note that catharsis-related responses such as crying are more likely to be regarded as therapeutically useful, e.g., see^20^), 79% of individuals reported experiencing sadness, 56% reported experiencing anxiousness, and 77% reported experiencing emotional or physical suffering. Among healthy individuals that were administered psilocybin, 31% of individuals reported experiencing strong or extreme fear and 22% reported that a significant portion or their *entire* psilocybin experience was characterized by anxiety or unpleasant psychological struggle^15^. Within a nationally representative sample, 40.9% of individuals with lifetime psychedelic use reported having a challenging psychedelic experience^19^. Cross-sectional surveys of psychedelic-induced challenging experiences^12^ and so-called “God encounter experiences”^21^ have also found that for some individuals (11% and 15%, respectively) these were the single most psychologically challenging experience of their life.

Although challenging experiences are sometimes described as ultimately beneficial or therapeutic^9,10,12,17,20,22^, these experiences can sometimes contribute to post-acute distress, functional impairment, and medical attention seeking (e.g.,^11,18,23–27^). For instance, among individuals with lifetime use of a classic psychedelic, 8.9% of individuals reported experiencing functional impairment for longer than one day, and 2.6% of individuals reported seeking medical or psychological assistance, following a challenging psychedelic experience^19^. There are also reports of the emergence of psychiatric diagnoses, suicidality, and harm to self and others during and after challenging psychedelic experiences^4,11,12^. Importantly, concerns about challenging experiences and their effects are commonly noted as a reason that health care providers^28–30^ and users^31–33^ are reluctant to suggest or receive treatment with classic psychedelics.

Several factors likely contribute to the intensity of challenging psychedelic experiences, including trait level neuroticism, preparedness for the psychedelic experience, and the setting in which the psychedelic is used^34–37^. Co-use of other pharmacological agents may also intensify or buffer against challenging experiences. For instance, relative to use of classic psychedelics alone, co-use of classic psychedelics with lithium and other mood stabilizers was associated with greater intensity of challenging experiences^19^ (in addition to medical complications, such as seizures^38,39^). Another study found a quadratic relationship between co-use of cannabis and classic psychedelics, such that (relative to use of a classic psychedelic alone) co-use of low dose cannabis was associated with less intense challenging experiences and co-use of large dose cannabis was associated with more intense challenging experiences^40^.

Co-use of 3,4-methylenedioxymethamphetamine (MDMA) with psilocybin (referred to as “hippy flipping”) and LSD (referred to as “candy flipping”) is one method that is reportedly used to reduce challenging experiences and enhance positive experiences^41,42^. MDMA, a potent serotonergic entactogen/empathogen, induces the release of serotonin, norepinephrine, dopamine, vasopressin, and oxytocin; dampens amygdala blood flow^43^; decreases feelings of fear and sadness^44,45^; and may increase positive feelings^43^, including love^46,47^, compassion^48,49^, and self-compassion^50,51^.

Several studies have reported on co-use of LSD/psilocybin and MDMA, with prevalence rates ranging from 8 to 52% among recreational drug users with lifetime LSD/psilocybin use^41,42,52–54^. Among polydrug users in the United Kingdom, participants reported co-using LSD and MDMA to improve the effect of LSD and to ease its aftereffects^41^. As one anecdotal report noted, “…when taken in conjunction…[MDMA] acts as a safety buffer and allows you to go a lot further than you normally would”^55^. Importantly, to date, only a single study has examined the effects of co-using MDMA alongside LSD in humans (with no studies on co-use alongside psilocybin). This was a recent double-blind placebo-controlled study^56^ that did not observe significant differences in acute experiences between LSD (100 µg) plus placebo relative to LSD (100 µg) plus MDMA (100 mg). Importantly, this study was conducted in a controlled setting and excluded individuals with a personal or family history of psychiatric disorders, which reduce the potential for LSD-related challenging experiences^10,12,22^ and thereby may have resulted in floor effects. Furthermore, the study examined only a single (medium–high) dose of MDMA and did not measure certain positive experiences (e.g., self-compassion, compassion, love, and gratitude) that may be impacted by MDMA. Finally, the study sample was small (*N* = 24), which increases the likelihood of Type II errors. Therefore, in an observational study, we further examined whether (relative psilocybin/LSD use alone) co-use of psilocybin/LSD and MDMA was associated with lower acute challenging experiences and increased acute positive experiences.

## Results

### Demographics and identification of covariates

The final sample included 698 individuals. For participant demographics, see Table 1. 342 individuals reported using LSD and 356 individuals reported using psilocybin during their experience. 27 individuals co-used psilocybin/LSD and MDMA (psilocybin + MDMA = 14; LSD + MDMA = 13). For further details regarding LSD/psilocybin dosage, see Fig. 2. For means and standard deviations for dependent variable and Kruskal Wallis tests (and post hoc Dunn’s tests), see Table 2.

**Table 1:** Demographics.

Demographic	Category	N(%)	*M(SD)*
Age^#^			30.18 (10.68)
Sex^#^	Female	199 (29.5)	
	Male	467 (69.2)	
	Other	9 (1.3)	
Nationality	United States	184 (26.4)	
	United Kingdom	183 (26.2)	
	Canada	41 (5.9)	
	Germany	34 (4.9)	
	Denmark	19 (2.7)	
	Other	237 (34.0)	
Employment	Full-time employment	281 (40.3)	
	Part-time employment	85 (12.2)	
	Retired	14 (2.0)	
	Student	223 (31.9)	
	Unemployed	72 (10.3)	
Education	Left school before age 16 without qualifications	15 (2.1)	
	Some high school/GCSE level (in UK)	46 (6.6)	
	High school diploma/A-level education (in UK)	110 (15.8)	
	Some university (or equivalent)	160 (22.9)	
	Bachelor’s degree (or equivalent)	197 (28.2)	
	Post-graduate degree (e.g., masters or doctorate)	147 (21.1)	
Lifetime psychiatric diagnosis (Yes)^#^	188 (26.9)		
Lifetime prescribed psychiatric medication (Yes)^#^	230 (34.1)		
Currently prescribed psychiatric medication (Yes)^#^	66 (9.8)		
Currently prescribed antidepressants (Yes)^#^	40 (6.1)		
Lifetime psychedelic use (Yes)^#^	607 (89.9)		
Lifetime psychedelic use (frequency)^#^	Never	68 (10.1)	
	Once	49 (7.3)	
	2–5 times	162 (24.0)	
	6–10 times	114 (16.9)	
	11–20 times	103 (15.3)	
	21–50 times	99 (14.7)	
	51–100 times	41 (6.1)	
	More than 100 times	39 (5.8)	

^#^Data available for 675 individuals.

**Table 2:** Descriptive data and comparison of dependent variables by MDMA use.

Variable	Sample	Mean (*SD*)	Statistic	*p*
Challenging experiences				
Challenging experience total (CEQ-Total)	Main effect		**6.98**	**0.030**
	No MDMA	18.83 (15.50)	–	–
	Low dose MDMA	9.18 (5.93)	**2.59**	**0.010**
	Medium–high dose MDMA	19.10 (12.06)	− 0.49	0.627
CEQ subscales				
Grief	Main effect		**13.04**	**0.001**
	No MDMA	22.60 (21.87)	–	–
	Low dose MDMA	6.44 (8.68)	**3.28**	**0.001**
	Medium–high dose MDMA	14.44 (19.30)	− 1.58	0.114
Fear	Main effect		**7.62**	**0.022**
	No MDMA	20.63 (22.57)	–	–
	Low dose MDMA	7.47 (8.12)	**2.19**	**0.028**
	Medium–high dose MDMA	27.00 (18.14)	− 1.64	0.102
Physical distress	Main effect		1.29	0.525
	No MDMA	20.92 (17.77)	–	–
	Low dose MDMA	19.47 (12.18)	–	–
	Medium–high dose MDMA	25.67 (16.84)	–	–
Insanity	Main effect		2.66	0.265
	No MDMA	16.77 (22.76)	–	–
	Low dose MDMA	8.44 (11.12)	–	–
	Medium–high dose MDMA	22.78 (22.65)	–	–
Isolation	Main effect		3.56	0.168
	No MDMA	20.11(24.27)	–	–
	Low dose MDMA	8.89 (13.25)	–	–
	Medium–high dose MDMA	17.22 (24.20)	–	–
Death	Main effect		**6.01**	**0.050**
	No MDMA	10.73 (23.21)		
	Low dose MDMA	0.00 (0.00)	**2.36**	**0.018**
	Medium–high dose MDMA	6.67 (9.85)	− 0.63	0.531
Paranoia	Main effect		0.97	0.065
	No MDMA	6.99 (14.42)	–	–
	Low dose MDMA	6.67 (15.89)	–	–
	Medium–high Dose MDMA	6.67 (14.98)	–	–
Positive experiences				
Self-compassion*	Main effect		4.12	0.128
	No MDMA	58.34 (34.71)	–	–
	Low dose MDMA	79.00 (36.23)	–	–
	Medium–high dose MDMA	49.43 (23.20)	–	–
Compassion*	Main effect		3.66	0.161
	No MDMA	60.24 (33.49)	–	–
	Low dose MDMA	82.57 (26.57)	–	–
	Medium–high dose MDMA	69.00 (21.57)	–	–
Gratitude*	Main effect		**11.35**	**0.003**
	No MDMA	58.06 (34.56)	–	–
	Low dose MDMA	91.43 (11.86)	− **2.80**	**0.005**
	Medium–high dose MDMA	34.43 (29.65)	1.81	0.070
Love*	Main effect		**6.50**	**0.039**
	No MDMA	59.38 (35.04)	–	–
	Low dose MDMA	90.43 (18.17)	− **2.53**	**0.011**
	Medium–high dose MDMA	59.00 (33.28)	0.25	0.804
Mystical-type experience total (MEQ-30)	Main effect		1.94	0.378
	No MDMA	57.76 (22.41)	–	–
	Low dose MDMA	53.37 (12.56)	–	–
	Medium–high dose MDMA	50.04 (23.40)	–	–
MEQ-30 subscales				
Mystical	Main effect		4.58	0.101
	No MDMA	60.03 (22.48)	–	–
	Low dose MDMA	45.93 (21.76)	–	–
	Medium–high dose MDMA	52.17 (20.41)	–	–
Positive mood	Main effect		3.35	0.187
	No MDMA	64.69 (22.56)	–	–
	Low dose MDMA	75.56 (13.44)	–	–
	Medium–high dose MDMA	58.15 (24.89)	–	–
Transcendence	Main effect		0.70	0.706
	No MDMA	47.64 (28.09)	–	–
	Low dose MDMA	39.26 (15.07)	–	–
	Medium–high dose MDMA	46.67 (28.87)	–	–
Ineffability	Main effect		0.81	0.667
	No MDMA	65.71 (30.14)	–	–
	Low dose MDMA	74.81 (18.49)	–	–
	Medium–high dose MDMA	57.78 (35.43)	–	–

* = Data only collected in Study 1. **Bold text** indicates *p* < 0.05. Effects for Low Dose MDMA and Medium–High Dose MDMA are relative to No MDMA.

MDMA use (none, low dose, and medium–high dose) was significantly associated with: (a) conscientiousness (*F* = 3.20, *p* = 0.041; individuals who co-used low dose MDMA were significantly lower than those who did not co-use MDMA); (b) openness (*F* = 3.23, *p* = 0.040; individuals who co-used medium–high dose MDMA were significantly lower than those who did not co-use MDMA); and psilocybin/LSD use in the following contexts (c) recreational/social (χ^2^ = 18.80, *p* < 0.001; more common among co-users of low and medium–high dose MDMA); (d) live singing (χ^2^ = 9.81, *p* = 0.007), (e) emotional support (χ^2^ = 9.38, *p* = 0.009), and (f) strangers (χ^2^ = 15.88, *p* < 0.001; higher among co-users of low dose MDMA relative to those who did not co-use MDMA). Correlation coefficients and VIFs were all below cutoffs (i.e., all *r* < 0.4 and all VIF < 5), indicating that multicollinearity was not of significant concern. These variables were therefore included in the primary analyses (see below) examining the relationship between co-MDMA use with psilocybin/LSD and acute challenging and positive experiences. See Supplementary Material (Supplementary Table 1) for a full list of analyses examining potential confounds.

### Primary analyses

#### Challenging experiences

Co-use of MDMA with psilocybin/LSD was associated with significant differences in total challenging experience (F(2,672) = 3.62, *p* = 0.031). Relative to psilocybin/LSD use alone, psilocybin/LSD + low dose MDMA was associated with significantly lower levels of total challenging experience, *t*(672) = 2.54, *p* = 0.011. There was no significant difference between psilocybin/LSD use alone and psilocybin/LSD + medium–high dose MDMA use, *t*(672) =  − 0.68, *p* = 0.498.

Examining group differences on CEQ subscales, co-use of MDMA with psilocybin/LSD was associated with significant differences in experiences of grief (F[2,672] = 4.64, *p* = 0.012) and fear (F[2,672] = 33.80, *p* = 0.023), but not physical distress (F[2,672] = 0.43, *p* = 0.654), insanity (F[2,672] = 1.30, *p* = 0.273), isolation (F[2,672] = 1.25, *p* = 0.287), death (F[2,672] = 2.42, *p* = 0.090), or paranoia (F[2,672] = 1.64, *p* = 0.196). Relative to psilocybin/LSD use alone, psilocybin/LSD + *low dose* MDMA was associated with significantly lower levels of grief and fear (*t*[672] = 2.83, *p* = 0.005; *t*[672] = 2.21, *p* = 0.027, respectively). There was no significant difference between psilocybin/LSD use alone and psilocybin/LSD + *medium–high* dose MDMA use for grief and fear (*t*[672] = 1.02, *p* = 0.310; *t*[672] =  − 1.61, *p* = 0.108, respectively).

#### Positive experiences

Co-use of MDMA with psilocybin/LSD was associated with higher levels of self-compassion, feelings of love, and experiences of gratitude (F(2,256) = 3.62, *p* = 0.028; F(2,256) = 3.97, *p* = 0.020; F(2,256) = 3.92, *p* = 0.021, respectively). Relative to psilocybin/LSD use alone, psilocybin/LSD + *low dose* MDMA was associated with greater feelings of self-compassion, love, and gratitude (*t*(256) =  − 2.61, *p* = 0.010; *t*(256) =  − 2.69, *p* = 0.008; *t*(256) =  − 2.12, *p* = 0.035, respectively), while psilocybin/LSD + medium–high dose MDMA was not (*t*(256) = 0.59, *p* = 0.557; *t*(256) = 0.79, *p* = 0.431; *t*(256) = 1.78, *p* = 0.076, respectively). Co-use of MDMA with psilocybin/LSD was not associated with significant differences in compassion (F(2,256) = 2.67, *p* = 0.071) or mystical-type experience (total score F[2,577] = 0.65, *p* = 0.524; mystical F[2,556] = 0.49, *p* = 0.613; positive mood F[2,577] = 2.13, *p* = 0.120; transcendence F[2,577] = 0.35, *p* = 0.703; and ineffability F[2,577] = 0.87, *p* = 0.421).

## Discussion

Psilocybin/LSD experiences can range from being profoundly positive to overwhelmingly challenging. Anecdotal reports indicate that individuals sometimes co-use MDMA to buffer against challenging experiences and enhance positive experiences associated with psilocybin/LSD^55^. To date, only a single study had examined the association between co-use of MDMA and psilocybin/LSD and acute subjective drug effects. Therefore, in a convenience sample, this study examined whether co-use of MDMA with psilocybin/LSD is associated with lower challenging experiences and higher positive experiences.

Controlling for potential confounds, co-use of MDMA (specifically low dose) with psilocybin/LSD was associated with lower levels of *total* challenging experiences, as well as grief and fear (measured by CEQ Total and CEQ subscales, respectively). These reductions in total challenging experience, fear, and grief are in line with research indicating that MDMA reduces experiences of sadness and fear^44,45^ and anecdotal reports regarding the effects of “hippy flipping” and “candy flipping”^41,55^. Although death-related challenging experiences were also significantly lower among individuals that co-used low dose MDMA and psilocybin/LSD, when controlling for potential confounds, co-use of MDMA was not associated with significant differences in death-related or other aspects of challenging experiences (i.e., physical distress, insanity, isolation, and paranoia). These non-significant results may be explained by: (1) co-MDMA use targeting affective/emotional systems over cognitive systems, explaining why emotions like fear and grief were altered, while having limited influence on more cognitively-dependent states like death and paranoia; (2) floor effects and high variability (i.e., ‘fear of death’ was low across groups, and the mean score for the low dose MDMA group was 0; ‘isolation’ and ‘insanity’ have large standard deviations); and/or (3) underpowered sample size for small-to-moderate effects in non-parametric analyses.

Regarding positive experiences, co-use of low dose MDMA (but not medium–high dose MDMA) with psilocybin/LSD was associated with enhanced feelings of self-compassion, love, and gratitude relative to psilocybin/LSD alone. These findings are in line with previously reported motivations for co-using MDMA with psilocybin/LSD^41^, as well as research indicating that MDMA (alone) may increase acute positive experiences (e.g.,^46,47^). We did not find significant differences between groups for mystical-type experiences (MEQ-30 total score or subscale scores) and compassion (single-item measure) suggesting that these experiences may be unaffected. However, it is noteworthy that (compared with LSD/psilocybin alone) co-use of low dose MDMA was associated with relatively higher mean scores for compassion and relatively lower mean scores for total mystical-type experience. Interestingly, while several MEQ-30 subscales (i.e., positive mood and ineffability) were descriptively higher in the group that co-used low dose MDMA, other subscales (i.e., mystical and transcendence) were descriptively lower in this group, suggesting a potentially complex relationship between co-use of MDMA and mystical-type experiences. Further research in larger samples is needed to causally elucidate these relationships.

We did not observe any significant differences between co-use of medium–high dose MDMA and the psilocybin/LSD alone groups for acute challenging or positive experiences. This dose-dependent relationship is similar to that previously observed for co-use of cannabis with classic psychedelics^40^, which found that while co-use of low dose cannabis was associated with lower challenging experiences, co-use of high dose cannabis was associated with greater total challenging experiences, fear, and grief. These null findings are also in line with a recently conducted placebo-controlled study in which (relative to LSD [100 µg] and placebo) co-administration of LSD with a medium–high dose of MDMA (100 mg) was not associated with significant differences in challenging or positive experiences^56^. While neither found statistically significant effects for co-use of medium–high dose MDMA, we caution against inferring that co-use of medium–high dose MDMA does *not* impact the acute psilocybin/LSD experience (i.e., the analyses fail to reject the null but *do not* provide evidence for the null hypothesis; for discussions, see^57–59^), especially given the relatively small sample sizes. Further studies with larger samples will remain necessary. Nonetheless, these findings suggest that the relationship between co-use of MDMA and LSD/psilocybin may be dose dependent and that further research with exact doses of psilocybin/LSD and MDMA are necessary to understand the potentially complex relationship between these substances.

Findings from this study suggest that co-use of low dose MDMA with psilocybin/LSD may buffer against negative or challenging experiences and enhance certain positive experiences. These findings may inform future clinical trial designs and provide early insights into recreational co-use of MDMA with psilocybin/LSD. ​Given the nontrivial presence of challenging experiences within clinical research (e.g.,^13^) and non-clinical (e.g.,^11,12,17,19,21^) administration/use of psilocybin/LSD, these findings suggest that co-administration of MDMA may help to mitigate such experiences, as well as post-acute distress, functional impairment, and medical attention seeking that is sometimes reported following challenging psychedelic experiences^11,18,23–27^).

Provided that pharmacokinetic and larger controlled studies confirm the present preliminary findings and establish the safety and feasibility of co-administering MDMA with psilocybin/LSD, individuals with elevated anxiety about challenging experiences and clinical presentations/profiles (e.g., individuals with elevated neuroticism^34^, avoidant attachment style^60^, borderline personality disorder^61,62^, poor therapeutic alliance^63^) at a greater risk of challenging experiences may benefit from MDMA co-administration. However, further research will be necessary to examine such speculative hypotheses. MDMA-attributed increases in positive experiences may also be particularly beneficial in specific therapeutic contexts, including couples-based treatment (e.g., see^64^), positive psychology interventions (which are often gratitude focused; e.g., see^65^), and group-based treatment/sessions^66,67^.

Importantly, addressing concerns about challenging experiences through potential co-administration of MDMA, may help to reduce anxiety and increase openness to psychedelic-assisted psychotherapy among health care providers^28–30^ and users^31–33^. Considering the unique mechanisms of action of MDMA and psilocybin/LSD and the growing preliminary support for their efficacy for specific psychiatric diagnoses (posttraumatic stress disorder^68^ and depression, anxiety, and alcohol use^1^, respectively), it is also possible co-administration might potentiate the potential efficacy of either compound alone. Contrarily, it remains unclear if challenging experiences are integral to the therapeutic process and mental health improvements–as has been reported by some^9,22^, leaving open the possibility that co-administration of MDMA may interfere with the therapeutic process.

### Limitations and future directions

The present study has considerable limitations including a small sample size, convenience sampling method, and uncontrolled design. The small sample size and potential floor effects may have contributed to null findings and a risk of Type 2 errors (i.e., false negatives). Additionally, given the exploratory nature of the present study and the limited power (due to the sample size and number of covariates included in the models), the present analyses were not corrected for multiple comparisons. Follow-up confirmatory studies are therefore needed to establish confidence in the replicability of the present findings. While the study did not use a controlled design (i.e., precise dosages are unknown, lack of random assignment, self-selected sample etc.), the use of a convenience sample bears some benefit to generalizability, as it is likely more reflective of “hippy-flipping” and “candy-flipping” in Western recreational users. The prospective recruitment and consistency in post-co-use data collection (day after use) are superior to other retrospective studies, which may be more confounded by time and memory-related effects. Additionally, the study examined and controlled for a wide range of potential confounds, including personality factors and the context in which LSD/psilocybin (with or without MDMA) were used. Use of psilocybin vs. LSD was also examined as a potential confound, providing preliminary support for the present effects generalizing across both psilocybin and LSD. Considering the sample largely consisted of psychedelic-experienced users of a particular demographic, further research is needed to determine whether these findings generalize to those who are psychedelic-naive and of other demographic status (e.g., minoritized individuals^69,70^). Additionally, the majority of the positive experiences (i.e., self-compassion, compassion, gratitude, and love) were measured using single non-validated items, limiting interpretation. Finally, information was not available regarding the exact timing of psilocybin/LSD and MDMA co-use or the MDMA dosage that was considered low, medium, or high (while some research identifies low dose MDMA as 50–75 mg^71^, other research identifies low dose MDMA as 30–49 mg^68^), which will be important for designing future controlled studies on co-administration of psilocybin/LSD and MDMA. Future studies are needed to confirm these findings utilizing larger sample sizes, healthy and clinical samples, validated psychometric instruments, and randomized controlled designs. Dose–response designs in which interactions between precise doses of MDMA and psilocybin/LSD (ranging from low to very high dosages) are administered, as well as interactions with individual traits and psychiatric diagnoses, may benefit clinical application and precision-based medicine.

## Methods

### Design and procedure

​​The present study examined the impact of co-use of MDMA and psilocybin/LSD (relative to psilocybin/LSD alone) on acute challenging and positive experiences. Data was collected as part of two online prospective surveys of individuals with upcoming plans to use a psychedelic substance in a naturalistic setting. Data unrelated to co-use of MDMA has previously been published from Study 1^36,40,72^ and Study 2^73–76^. Study designs were nearly identical and therefore data were collapsed across the two studies. The studies were approved by the Imperial College London’s Research Ethics Committee and Joint Research Compliance Office and were conducted in accordance with principles of Good Clinical Practice.

Eligibility criteria for both studies were as follows: (1) 18 years or older; (2) ability to read/write English; and (3) intention to use a psychedelic substance (e.g., psilocybin/magic mushrooms/truffles, MDMA, LSD/ 1-propionyl-lysergic acid diethylamide (1P-LSD), ayahuasca, *N*,*N*-Dimethyltryptamine (DMT), 5-methoxy-*N*,*N*-dimethyltryptamine (5-MeO-DMT), mescaline, 2,5-dimethoxy-4-bromophenethylamine (2C-B), salvia divinorum, iboga/ibogaine). Individuals were included in the present manuscript if they used psilocybin or LSD alone or co-used psilocybin or LSD and MDMA during their experience. Individuals were excluded from the present analyses if they used substances other than (a) psilocybin/LSD alone *or* (b) psilocybin/LSD *and* MDMA during their experience.

Participants were recruited using online advertisements, postings on Facebook, Twitter, and email newsletters, and online forums (e.g., Reddit). Interested participants reviewed study details and provided informed consent online along with their email address. Surveys were subsequently sent via email depending upon the date the participant intended to use a psychedelic. Surveys relevant to the present manuscript were administered seven days prior to the planned psychedelic experience and 1 day after the planned psychedelic experience. The following data was collected prior to participants’ psychedelic experience: demographics (age, sex, nationality, employment, and education); personality (Extraversion, Agreeableness, Conscientiousness, Emotional Stability, and Openness to Experiences; measured via the Ten Item Personality Measure [TIPI]^77^); self-reported psychiatric history (previous and/or current use of psychiatric medications, lifetime psychiatric disorder); and lifetime psychedelic use (frequency).

**Figure 1:**
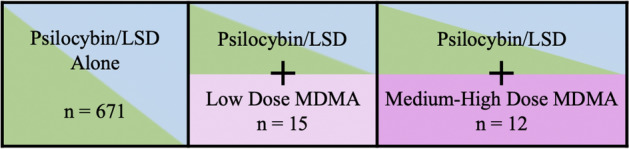
Categorization by psilocybin/LSD and co-use of MDMA by dose.

Following their psychedelic experience, participants identified the psychedelic they used and the dose they used: (1) Low dose (e.g., < 50 μg LSD); (2) Moderate dose (e.g., 51–100 μg LSD); (3) High dose (e.g., 101–200 μg LSD); (4) Very high dose (e.g., 201–300 μg LSD); and (5) Extremely high dose (> 300 μg LSD) (Fig. 1). Participants were also asked whether they co-used MDMA during their experience and the dose of MDMA they used: (1) None; (2) Low; (3) Medium; and (4) High. Participants were asked to identify (yes/no) whether they had their psychedelic experience in specific settings (retreat, reactional/social, and/or therapeutic) and whether their experience featured the following elements: music; live singing; emotional support; sense of threat; strangers, and/or disruption. Finally, relating to their psychedelic use 1 day prior, participants completed measures of acute challenging and positive experiences (see ‘Measures’ section below). All data was collected using the online ‘Psychedelic Survey’ platform (https://www.psychedelicsurvey.com).

### Measures

#### Challenging experiences

Challenging experiences were measured using the Challenging Experience Questionnaire (CEQ^10^), a 26-item scale developed to characterize acute adverse experiences occasioned by psychedelic substances^10^. Subscales of the CEQ measure grief (6 items), fear (5 items), physical distress (5 items), insanity (3 items), isolation (3 items), death (2 items), and paranoia (2 items). Reflecting on a particular psychedelic experience, items are rated on a six-point Likert scale ranging from 0 (none; not at all) to 5 (extreme [more than ever before in my life]). In line with past research^20^, total challenging experiences were scored by calculating the mean of all 26 items multiplied by 20 to provide a score ranging from 0 to 100. Similarly, subscales were scored by calculating the mean for each subscale multiplied by 20. The CEQ has been utilized in both non-clinical^10,12,20,36^ and clinical^7,21^ studies of classic psychedelic experiences.

#### Positive experiences

##### Self-compassion, compassion, love, and gratitude

Positive experiences of self-compassion, compassion, gratitude, and love were each measured using individual self-constructed items. Reflecting on their psychedelic experience, participants rated each item on a visual analogue scale from 0 (No/not more than usual) to 100 (Yes/very much more than usual). Items were as follows: (1) self-compassion (“I felt compassion towards myself”); (2) compassion (“I felt compassion towards others”); (3) gratitude (“I felt a great sense of gratitude”); and (4) love (“I felt a great sense of love”). These items were only collected in Study 1 (*n* = 282).

#### Mystical experience

Mystical-type experiences were measured using the revised Mystical Experience Questionnaire (MEQ-30^78^). The MEQ-30 is a 30-item measure of mystical effects of classic psychedelics composed of four factors: (1) mystical (i.e., unity, noetic quality, and sacredness; 15 items); (2) deeply felt positive mood (6 items); (3) transcendence of time and space (6 items); and (4) ineffability/paradoxicality (3 items). Items are rated on a six-point Likert scale ranging from 0 (none/not at all) to 5 (extreme [more than any other time in my life]). Total mystical experience was scored by calculating the mean of all 30 items multiplied by 20 to provide a score ranging from 0 to 100. Subscale scores were similarly calculated using the relevant items. The MEQ-30 has been used widely in both non-clinical (e.g.,^36,79,80^) and clinical samples (e.g.,^81–83^).

### Statistical analyses

**Figure 2:**
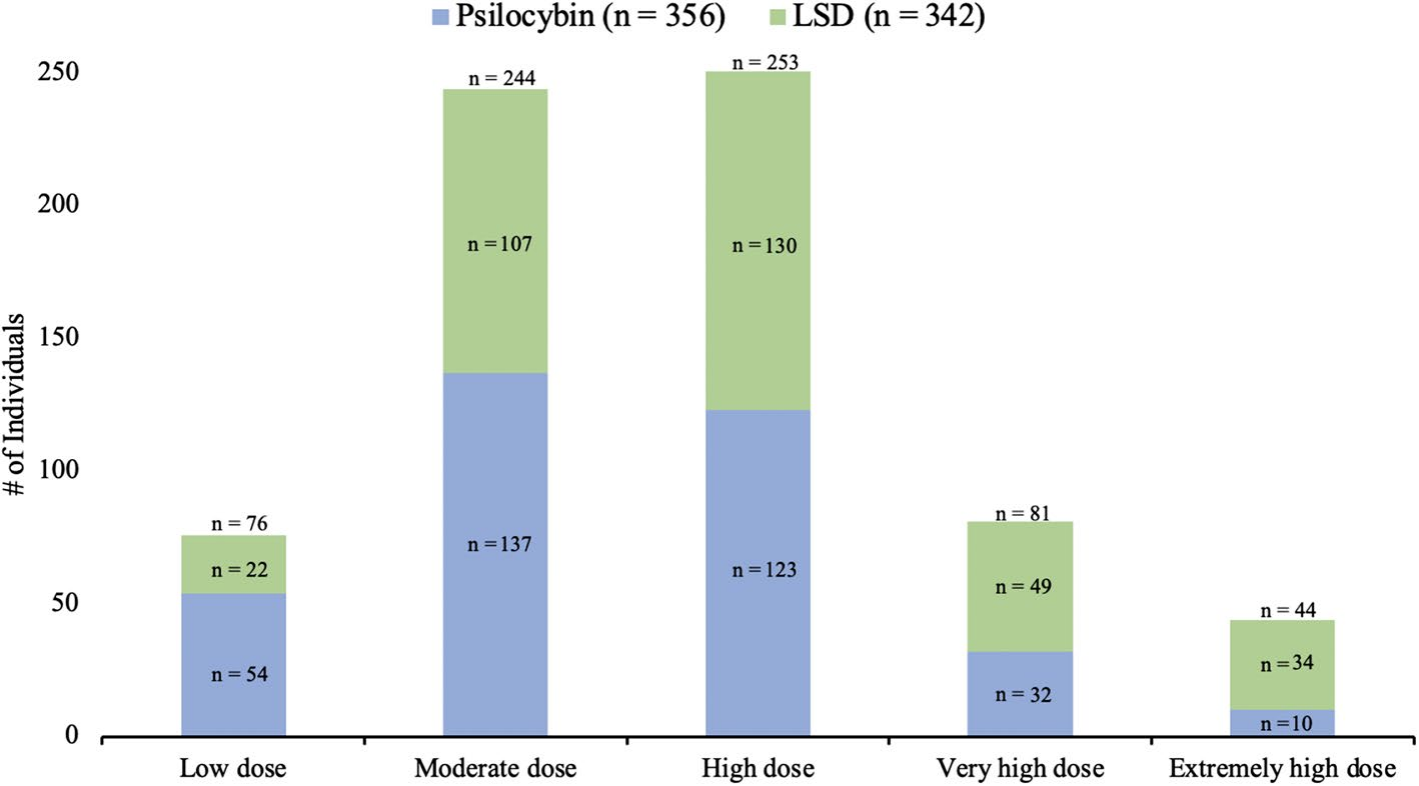
Distribution of psilocybin/LSD use by dose.

Only one individual reported co-using psilocybin/LSD with high dose MDMA, therefore medium and high dose were collapsed into one category. Co-use of MDMA was categorized as either none (0), low (1), or medium–high (2), as shown in Fig. 2. We examined whether co-use of MDMA (none, low dose, and medium–high dose) with psilocybin/LSD predicted the intensity of participants’ challenging (total challenging experience [CEQ Total], grief, fear, physical distress, insanity, isolation, death, and paranoia [CEQ subscales]) and positive (love, gratitude, compassion, self-compassion, and mystical-type experience [MEQ-30 total score and mystical, positive mood, transcendence, and ineffability subscales]) experiences. All dependent variables were examined via Q-Q plots, histograms, and statistical analyses (i.e., Kolmogorov–Smirnov and Shapiro Wilk tests) and were found to be non-normally distributed (e.g., all Kolmogorov–Smirnov and Shapiro Wilk tests were *p* < 0.001). Therefore, we conducted a series of preliminary Kruskal–Wallis tests (without covariates). When these main effects were significant we then conducted Dunn’s post-hoc tests to compare psilocybin/LSD without MDMA against: (a) psilocybin/LSD + low dose MDMA; and (b) psilocybin/LSD + medium–high dose MDMA.

Based on past research^36,40,84,85^, the following variables were examined as potential confounding variables: age, sex, lifetime previous psychedelic use (yes/no), lifetime previous psychedelic use (frequency), lifetime psychiatric diagnosis, previous use of psychiatric medications, current use of psychiatric medications, current use of antidepressant medication, psilocybin or LSD use for experience, psilocybin/LSD dose, personality (Extraversion, Agreeableness, Conscientiousness, Emotional Stability, and Openness to Experiences; measured via the TIPI^77^), and setting (retreat, recreational/social, or therapeutic, presence of music, live singing, emotional support, a threat, strangers, and/or disruption). A series of statistical tests (ANOVAS for continuous variables and chi-squared tests for binary variables) were performed where MDMA dose was treated as the independent variable and potential confounds were included as the dependent variable. Variables that were significantly associated with MDMA dose (*p* < 0.05) were identified as potential confounds and were included as covariates in the primary analyses. Multicollinearity among the selected confounders were examined by calculating Pearson correlation coefficients (cut-off: *r* > 0.4) and variance inflation factors (VIFs; cut-off ≥ 5).

Quade nonparametric ANCOVAs were conducted wherein MDMA dose was the independent variable, acute experience measures were the dependent variable, and potential confounds were included as covariates. Post-hoc analyses were performed for significant group differences to determine if low dose and/or medium–high dose MDMA were responsible for significant effects. All analyses were conducted in SPSS (Version 28) and the threshold for statistical significance was set at *p* < 0.05, two-tailed.

### Supplementary Information

Supplementary Information.

## Data Availability

The data that support the findings of this study are available from the corresponding author, RJZ, upon reasonable request.

## References

[1] Schimmel N (2022). Psychedelics for the treatment of depression, anxiety, and existential distress in patients with a terminal illness: A systematic review. Psychopharmacol..

[2] Nutt DJ (2010). Drug harms in the UK: A multicriteria decision analysis. Lancet.

[3] Schlag AK (2022). Adverse effects of psychedelics: From anecdotes and misinformation to systematic science. J. Psychopharmacol..

[4] Zeifman RJ (2021). On the relationship between classic psychedelics and suicidality: A systematic review. ACS Pharmacol. Transl. Sci..

[5] Zeifman RJ (2022). Decreases in suicidality following psychedelic therapy: A meta-analysis of individual patient data across clinical trials. J. Clin. Psychiatry.

[6] Barrett FS, Griffiths RR (2018). Classic hallucinogens and mystical experiences: Phenomenology and neural correlates. Curr. Top. Behav. Neurosci..

[7] Roseman L (2018). Quality of acute psychedelic experience predicts therapeutic efficacy of psilocybin for treatment-resistant depression. Front. Pharmacol..

[8] Ona G (2018). Inside bad trips: Exploring extra-pharmacological factors. J. Psychedelic Stud..

[9] Johnstad PG (2021). Day trip to hell: A mixed methods study of challenging psychedelic experiences. J. Psychedelic Stud..

[10] Barrett FS (2016). The challenging experience questionnaire: Characterization of challenging experiences with psilocybin mushrooms. J. Psychopharmacol..

[11] Bremler, R. et al. Focusing on the negative: Cases of long-term negative psychological responses to psychedelics. *PsyArXiv* (2023). 10.31234/osf.io/yzmcj 10.1038/s41598-023-41145-x PMC10519946 37749109

[12] Carbonaro TM (2016). Survey study of challenging experiences after ingesting psilocybin mushrooms: Acute and enduring positive and negative consequences. J. Psychopharmacol..

[13] Davis AK (2021). Effects of psilocybin-assisted therapy on major depressive disorder: A randomized clinical trial. JAMA Psychiat..

[14] Garcia-Romeu A (2020). Persisting reductions in cannabis, opioid, and stimulant misuse after naturalistic psychedelic use: An online survey. Front. Psychiatry.

[15] Griffiths RR (2006). Psilocybin can occasion mystical-type experiences having substantial and sustained personal meaning and spiritual significance. Psychopharmacol..

[16] Holze F (2023). Lysergic acid diethylamide–assisted therapy in patients with anxiety with and without a life-threatening illness: A randomized, double-blind, placebo-controlled phase II study. Biol. Psychiatry.

[17] Lake S, Lucas P (2023). The Canadian psychedelic survey: Characteristics, patterns of use, and access in a large sample of people who use psychedelic drugs. Psychedelic Med.

[18] Larsen JK (2016). Neurotoxicity and LSD treatment: A follow-up study of 151 patients in Denmark. Hist. Psychiatry.

[19] Simonsson O (2023). Prevalence and associations of challenging, difficult or distressing experiences using classic psychedelics. J. Affect. Disord..

[20] Roseman L (2019). Emotional breakthrough and psychedelics: Validation of the emotional breakthrough inventory. J. Psychopharmacol..

[21] Griffiths RR (2019). Survey of subjective" God encounter experiences": Comparisons among naturally occurring experiences and those occasioned by the classic psychedelics psilocybin, LSD, ayahuasca, or DMT. PLoS ONE.

[22] Gashi L (2021). Making “bad trips” good: How users of psychedelics narratively transform challenging trips into valuable experiences. Int. J. Drug Policy.

[23] Barber G (2022). A case of prolonged mania, psychosis, and severe depression after psilocybin use: implications of increased psychedelic drug availability. Am. J. Psychiatry.

[24] Bouso JC (2022). Adverse effects of ayahuasca: Results from the Global Ayahuasca Survey. PLOS Glob. Public Health.

[25] Cohen S (1960). Lysergic acid diethylamide: Side effects and complications. J. Nerv. Ment. Dis..

[26] Durante Í (2020). Risk assessment of ayahuasca use in a religious context: Self-reported risk factors and adverse effects. Braz. J. Psychiatry.

[27] Strassman RJ (1984). Adverse reactions to psychedelic drugs. A review of the literature. J. Nerv. Ment. Dis..

[28] Beaussant Y (2020). Defining the roles and research priorities for psychedelic-assisted therapies in patients with serious illness: Expert clinicians' and investigators' perspectives. J. Palliat. Med..

[29] Niles H (2021). Palliative care provider attitudes toward existential distress and treatment with psychedelic-assisted therapies. BMC Palliat. Care.

[30] Reynolds LM (2021). Cancer healthcare workers’ perceptions toward psychedelic-assisted therapy: A preliminary investigation. Int. J Enviro. Res. Public Health.

[31] Clifton JM (2022). Psilocybin use patterns and perception of risk among a cohort of Black individuals with opioid use disorder. J. Psychedelic Stud..

[32] Corrigan K (2022). Psychedelic perceptions: Mental health service user attitudes to psilocybin therapy. Ir. J. Med. Sci..

[33] Winstock, A. R. et al. *Global drug survey (GDS) 2019 key findings report*, 16 May. London: Global Drug Survey (2019). https://issuu.com/globaldrugsurvey/docs/gds2019_key_findings_report_may_16_

[34] Barrett FS (2017). Neuroticism is associated with challenging experiences with psilocybin mushrooms. Pers. Individ. Differ..

[35] Carhart-Harris RL (2018). Psychedelics and the essential importance of context. J. Psychopharmacol..

[36] Haijen EC (2018). Predicting responses to psychedelics: A prospective study. Front. Pharmacol..

[37] Aday JS (2021). Predicting reactions to psychedelic drugs: A systematic review of states and traits related to acute drug effects. ACS Pharmacol. Transl. Sci.

[38] Nayak SM (2021). Classic psychedelic coadministration with lithium, but not lamotrigine, is associated with seizures: An analysis of online psychedelic experience reports. Pharmacopsychiatry.

[39] Simonsson O (2022). Prevalence and associations of classic psychedelic-related seizures in a population-based sample. Drug Alcohol Depend..

[40] Kuc J (2022). Psychedelic experience dose-dependently modulated by cannabis: Results of a prospective online survey. Psychopharmacol..

[41] Boys A (2001). Understanding reasons for drug use amongst young people: A functional perspective. Health Educ. Res..

[42] Licht CL (2012). Simultaneous polysubstance use among Danish 3,4-methylenedioxymethamphetamine and hallucinogen users: Combination patterns and pro- posed biological bases. Hum. Psychopharmacol..

[43] Carhart-Harris RL (2015). The effects of acutely administered 3, 4-methylenedioxymethamphetamine on spontaneous brain function in healthy volunteers measured with arterial spin labeling and blood oxygen level–dependent resting state functional connectivity. Biol. Psychiatry.

[44] Dolder PC (2018). Direct comparison of the acute subjective, emotional, autonomic, and endocrine effects of MDMA, methylphenidate, and modafinil in healthy subjects. Psychopharmacol..

[45] Dumont GJH, Verkes RJ (2006). A review of acute effects of 3, 4-methylenedioxymethamphetamine in healthy volunteers. J. Psychopharmacol..

[46] Bedi G (2010). Is ecstasy an “empathogen”? Effects of±3, 4-methylenedioxymethamphetamine on prosocial feelings and identification of emotional states in others. Biol. Psychiatry.

[47] Studerus E (2010). Psychometric evaluation of the altered states of consciousness rating scale (OAV). PLoS ONE.

[48] Feduccia AA, Mithoefer MC (2018). MDMA-assisted psychotherapy for PTSD: Are memory reconsolidation and fear extinction underlying mechanisms?. Prog. Neuro-Psychopharmacol. Biol Psychiatry.

[49] Passie T (2018). The early use of MDMA (‘Ecstasy’) in psychotherapy (1977–1985). Drug Sci. Policy Law.

[50] Kamboj SK (2015). Recreational 3, 4-methylenedioxy-N-methylamphetamine (MDMA) or ‘ecstasy’and self-focused compassion: Preliminary steps in the development of a therapeutic psychopharmacology of contemplative practices. J. Psychopharmacol..

[51] Kamboj SK (2018). Additive effects of 3, 4-methylenedioxymethamphetamine (MDMA) and compassionate imagery on self-compassion in recreational users of ecstasy. Mindfulness.

[52] Grov C (2009). Polydrug use among club-going young adults recruited through time-space sampling. Subst. Use Misuse.

[53] Sanders B (2008). Multiple drug use and polydrug use amongst homeless traveling youth. J. Ethn. Subst. Abuse.

[54] Winstock AR (2001). Drugs and the dance music scene: A survey of current drug use patterns among a sample of dance music enthusiasts in the UK. Drug Alcohol Depend..

[55] Schechter MD (1998). Candyflipping': Synergistic discriminative effect of LSD and MDMA. Eur. J. Pharmacol..

[56] Straumann, I. et al. (2023). Acute effects of MDMA and LSD co-administration in a double-blind placebo-controlled study in healthy participants. *Neuropsychopharmacol.* (2023). Advance online publication. 10.1038/s41386-023-01609-0 10.1038/s41386-023-01609-0 PMC10584820 37258715

[57] Altman DG, Bland JM (1995). Statistics notes: Absence of evidence is not evidence of absence. BMJ.

[58] Lakens D (2020). Improving inferences about null effects with Bayes factors and equivalence tests. J. Gerontol. BPsychol Sci. Soc. Sci..

[59] Rogers, J. L. et al. (1993). Using significance tests to evaluate equivalence between two experimental groups. *Psychol. Bull. ****113***, 553–565 (1993). 10.1037/0033-2909.113.3.553 8316613

[60] Stauffer CS (2020). Psilocybin-assisted group therapy and attachment: Observed reduction in attachment anxiety and influences of attachment insecurity on the psilocybin experience. ACS Pharmacol. Transl. Sci..

[61] Traynor JM (2022). MDMA-assisted psychotherapy for borderline personality disorder. Focus.

[62] Zeifman RJ, Wagner AC (2020). Exploring the case for research on incorporating psychedelics within interventions for borderline personality disorder. J. Contextual Behav. Sci..

[63] Murphy R (2022). Therapeutic alliance and rapport modulate responses to psilocybin assisted therapy for depression. Front. Pharmacol..

[64] Wagner AC (2021). Couple therapy with MDMA—Proposed pathways of action. Front. Psychol..

[65] Griffiths RR (2018). Psilocybin-occasioned mystical-type experience in combination with meditation and other spiritual practices produces enduring positive changes in psychological functioning and in trait measures of prosocial attitudes and behaviors. J. Psychopharmacol..

[66] Kettner H (2021). Psychedelic communitas: Intersubjective experience during psychedelic group sessions predicts enduring changes in psychological wellbeing and social connectedness. Front. Pharmacol..

[67] Trope A (2019). Psychedelic-assisted group therapy: A systematic review. J. Psychoactive Drugs.

[68] Smith KW (2022). MDMA-assisted psychotherapy for treatment of posttraumatic stress disorder: A systematic review with meta-analysis. J. Clin. Pharmacol..

[69] Jones GM, Nock MK (2022). Race and ethnicity moderate the associations between lifetime psychedelic use (MDMA and psilocybin) and psychological distress and suicidality. Sci. Rep..

[70] Michaels TI (2018). Inclusion of people of color in psychedelic-assisted psychotherapy: A review of the literature. BMC Psychiatry.

[71] Bouso JC (2008). MDMA-assisted psychotherapy using low doses in a small sample of women with chronic posttraumatic stress disorder. J. Psychoactive Drugs.

[72] Close JB (2020). Psychedelics and psychological flexibility–Results of a prospective web-survey using the Acceptance and Action Questionnaire II. J. Contextual Behav. Sci..

[73] Peill JM (2022). Validation of the Psychological Insight Scale: A new scale to assess psychological insight following a psychedelic experience. J. Psychopharmacol..

[74] Spriggs MJ (2021). Positive effects of psychedelics on depression and wellbeing scores in individuals reporting an eating disorder. Eat. Weight Disord..

[75] Watts R (2022). The Watts Connectedness Scale: A new scale for measuring a sense of connectedness to self, others, and world. Psychopharmacol..

[76] Zeifman RJ (2020). Post-psychedelic reductions in experiential avoidance are associated with decreases in depression severity and suicidal ideation. Front. Psychiatry.

[77] Gosling SD (2003). A very brief measure of the Big-Five personality domains. J. Res. Pers..

[78] MacLean KA (2012). Factor analysis of the mystical experience questionnaire: A study of experiences occasioned by the hallucinogen psilocybin. J. Sci. Study Relig..

[79] Agin-Liebes G (2022). Prospective examination of the therapeutic role of psychological flexibility and cognitive reappraisal in the ceremonial use of ayahuasca. J. Psychopharmacol..

[80] Davis AK (2020). Psychological flexibility mediates the relations between acute psychedelic effects and subjective decreases in depression and anxiety. J. Contextual Behav. Sci..

[81] Ross S (2016). Rapid and sustained symptom reduction following psilocybin treatment for anxiety and depression in patients with life-threatening cancer: A randomized controlled trial. J. Psychopharmacol..

[82] Griffiths RR (2016). Psilocybin produces substantial and sustained decreases in depression and anxiety in patients with life-threatening cancer: A randomized double-blind trial. J. Psychopharmacol..

[83] Zeifman RJ (2023). How does psilocybin therapy work? An exploration of experiential avoidance as a putative mechanism of change. J. Affect. Disord..

[84] Erritzoe D (2019). Recreational use of psychedelics is associated with elevated personality trait openness: Exploration of associations with brain serotonin markers. J. Psychopharmacol..

[85] Weiss B (2021). Examining psychedelic-induced changes in social functioning and connectedness in a naturalistic online sample using the five-factor model of personality. Front. Psychol..

