# Supplementary material for: Co-use of MDMA with psilocybin/LSD may buffer against challenging experiences and enhance positive experiences
Source: Sci Rep. 2023 Aug 22;13:13645. doi: 10.1038/s41598-023-40856-5 (PMC10444769; doi:10.1038/s41598-023-40856-5)
Supplement: Supplementary file 1 — Supplementary Information. [file 41598_2023_40856_MOESM1_ESM.docx]

**Supplementary Material**

**Supplementary Table 1.** Potential Confounding Variables Predicting Co-Use of MDMA

| Variable | Mean (*SD*) or % | F | *p* |
| --- | --- | --- | --- |
| Age |  | 0.27 | .764 |
| Sex* |  | 0.29 | .866 |
| Lifetime Psychedelic Use (Frequency) |  | 1.58 | .207 |
| Lifetime Psychedelic Use (Yes/No) |  | 3.23 | .199 |
| Psilocybin vs. LSD Use |  | 0.04 | .981 |
| Psilocybin/LSD Dose |  | 0.75 | .473 |
| **Psychiatric History** | | | |
| Psychiatric Diagnosis (Yes/NO) |  | 0.66 | .721 |
| Past Use of Psychiatric Medication |  | 1.27 | .531 |
| Current Use of Psychiatric Medication |  | 1.52 | .468 |
| Current Use of Antidepressants |  | 2.34 | .311 |
| **Personality** | | | |
| Extraversion |  | 0.69 | .504 |
| Agreeableness |  | 0.18 | .839 |
| Conscientiousness |  | **3.20** | **.041** |
| No MDMA | 8.84 (2.26) |  |  |
| Low Dose MDMA | 7.64 (2.17) |  |  |
| Medium-High Dose MDMA | 9.91 (2.43) |  |  |
| Emotional Stability |  | 0.30 | .745 |
| Openness |  | **3.23** | **.040** |
| No MDMA | 9.84 (2.31) |  |  |
| Low Dose MDMA | 10.36 (2.65) |  |  |
| Medium-High Dose MDMA | 11.55 (2.51) |  |  |
| **Context** | | | |
| Retreat |  | 3.58 | .167 |
| Therapeutic |  | 2.59 | .274 |
| Recreational/Social |  | **18.80** | **<.001** |
| No MDMA | 18.2 |  |  |
| Low Dose MDMA | 53.3 |  |  |
| Medium-High Dose MDMA | 50.0 |  |  |
| Live Singing |  | **9.81** | **.007** |
| No MDMA | 12.5 |  |  |
| Low Dose MDMA | 40.0 |  |  |
| Medium-High Dose MDMA | 16.7 |  |  |
| Music |  | 2.48 | .290 |
| Emotional Support |  | **9.38** | **.009** |
| No MDMA | 47.8 |  |  |
| Low Dose MDMA | 80.0 |  |  |
| Medium-High Dose MDMA | 75.0 |  |  |
| Strangers |  | **15.88** | **<.001** |
| No MDMA | 19.6 |  |  |
| Low Dose MDMA | 40.0 |  |  |
| Medium-High Dose MDMA | 33.3 |  |  |
| Disruption |  | 3.41 | .182 |
| Threat |  | 1.18 | .554 |

*Due to the limited sample that identified as “Other”, only male and female were included in this analysis. **Bold text** indicates *p* < .05.
